# High SARS-CoV-2 infection rate in children unvaccinated with COVID-19 vaccine in Changzhou, China, shortly after lifting zero-COVID-19 policy in December 2022

**DOI:** 10.1186/s12879-024-09445-3

**Published:** 2024-06-05

**Authors:** Jie Tang, Yong Wang, Weiqin Lu, Zhihong Gao, Mingfeng Xu, Lin Wu, Jianhua Jin

**Affiliations:** 1 Department of Obstetrics and Gynecology, Wujin Hospital Affiliated with Jiangsu University, Changzhou, Jiangsu 213017 China; 2 Department of Obstetrics and Gynecology, The Wujin Clinical College of Xuzhou Medical University, Changzhou, Jiangsu 213017 China; 3 Department of Clinical Laboratory, Wujin People’s hospital, Changzhou, Jiangsu 213017 China; 4 Department of Respiratory Diseases, Changzhou Hospital of Traditional Chinese Medicine, Changzhou, Jiangsu 213003 China; 5 Department of Pediatrics, Wujin People’s hospital, Changzhou, Jiangsu 213017 China; 6 Department of Infection Management, Changzhou Hospital of Traditional Chinese Medicine, Changzhou, Jiangsu 213003 China; 7 Department of Oncology, Wujin Hospital Affiliated with Jiangsu University, Changzhou, Jiangsu 213017 China

**Keywords:** Antibody to SARS-CoV-19, Seropositivity, Children, COVID-19 vaccination, Discontinuation of zero-COVID-19 policy

## Abstract

**Background:**

China experienced an overwhelming COVID-19 pandemic from middle December 2022 to middle January 2023 after lifting the zero-COVID-19 policy on December 7, 2022. However, the infection rate was less studied. We aimed to investigate the SARS-CoV-2 infection rate in children shortly after discontinuation of the zero-COVID-19 policy.

**Methods:**

From February 20 to April 10, 2023, we included 393 children aged 8 months to less than 3 years who did not receive COVID-19 vaccination and 114 children aged 3 to 6 years who received inactivated COVID-19 vaccines based on the convenience sampling in this cross-sectional study. IgG and IgM antibodies against nucleocapsid (N) and subunit 1 of spike (S1) of SARS-CoV-2 (anti-N/S1) were measured with commercial kits (Shenzhen YHLO Biotech, China).

**Results:**

Of the 393 unvaccinated children (1.5 ± 0.6 years; 52.2% boys), 369 (93.9%) were anti-N/S1 IgG positive. Of the 114 vaccinated children (5.3 ± 0.9 years; 48.2% boys), 112 (98.2%) were anti-N/S1 IgG positive. None of the unvaccinated or vaccinated children was anti-N/S1 IgM positive. The median IgG antibody titers in vaccinated children (344.91 AU/mL) were significantly higher than that in unvaccinated children (42.80 AU/mL) (*P* < 0.0001). The positive rates and titers of anti-N/S1 IgG had no significant difference between boys and girls respectively.

**Conclusion:**

Vast majority of children were infected with SARS-CoV-2 shortly after ending zero-COVID-19 policy in China. Whether these unvaccinated infected children should receive COVID-19 vaccine merits further investigation.

## Background

Coronavirus disease 2019 (COVID-19), caused by severe acute respiratory syndrome coronavirus 2 (SARS-CoV-2), is still an important public health issue [1–3], although the World Health Organization (WHO) declared on May 5, 2023 that COVID-19 is no longer a global health emergency [4]. Since December 2020, several types of COVID-19 vaccines, including mRNA coding for spike protein of SARS-CoV-2, inactivated SARS-CoV-2, recombinant non-replicating adenovirus containing S gene of SARS-CoV-2, or recombinant S protein of SARS-CoV-2, have been used to prevent transmission of SARS-CoV-2 [5–9], although the protection duration is limited [10]. Nevertheless, breakthrough infection of SARS-CoV-2 appeared to be attenuated in persons immunized with COVID-19 vaccines, because the proportion of severe COVID-19 and the mortality of COVID-19 are substantially declined in the vaccinees [9, 11]. Therefore, COVID-19 vaccination plays an important role in preventing transmission of SARS-CoV-2 and reducing the mortality of COVID-19.

COVID-19 vaccination was initially implemented in adults at the age of equal to and over 18 years [6, 7], and then extended to children at the age of over three years. China started to vaccinate children and adolescents from July 2021 [12]. However, children under the age of three years are not recommended be vaccinated, and thus they are not eligible for COVID-19 vaccination. As time goes on, these children will become eligible for COVID-19 vaccination. Whether these children require COVID-19 vaccination is not ascertained, because it is unclear whether these children experienced natural SARS-CoV-2 infection, which can be determined by testing antibodies to SARS-CoV-2.

China had taken the extremely stringent comprehensive intervention measures named the zero-COVID-19 policy to prevent the transmission of SARS-CoV-2 after the initial outbreak of COVID-19 occurred in Wuhan city in early 2020 [13, 14]. Therefore, during the first three years of the pandemic COVID-19, only sporadic clusters of COVID-19 cases occurred in a few cities in mainland China and the transmission of SARS-CoV-2 was interrupted within a few weeks. The zero-COVID-19 policy was discontinued on December 7, 2022 [15]. From middle of December 2022 to middle of January 2023, China encountered an overwhelming number of COVID-19 cases. Measurement of antibodies to ORF8 of SARS-CoV-2 in 1500 patients aged 1–99 years during January 5 to 14, 2023 showed that 80.7% of them were infected after ending the zero-COVID-19 policy [16]. In the present serosurvey, we measured the antibodies to SARS-CoV-2 in children under three years age to estimate the infection rate of SARS-CoV-2 after discontinuation of the zero-COVID-19 policy.

## Materials and methods

### Design and study subjects

This was a cross-sectional seroepidemiological survey in children who were not vaccinated against COVID-19. In China, COVID-19 vaccination is not recommended for children aged < 3 years. In addition, attributed to the stringent zero-COVID-19 policy implemented in mainland China, no cases of COVID-19 among children in Changzhou city were defined before the discontinuation of zero-COVID-19 policy based on the epidemiologic data daily disclosed by the central government as well as the provincial and local health authorities. Thus, children under 3 years old were eligible for the study. Children under 8 months age were excluded because they were likely to have maternally derived anti-SARS-CoV-2 IgG, because most of women at childbearing ages received COVID-19 vaccines composed of inactivated SARS-CoV-2. Considering that the pandemic outbreak of COVID-19 in mainland China occurred from middle December 2022 to middle January 2023 [17] and that determination of SARS-CoV-2 infection by anti-SARS-CoV-2 IgG measurement is most sensitive after 4 weeks following symptom onset [18], we determined to collect blood samples from February 20 to April 10, 2023. Based on the convenience sampling, the children who visited Wujin People’s Hospital for various reasons including routine health examinations together with their parents or guardians and required peripheral venous blood for laboratory tests were included in this survey. Blood samples were taken by venipuncture. After the necessary laboratory tests such as blood routine tests and clinical biochemistry, residual serum or plasma samples were collected and stored at − 30 °C.

Although it was reported that that 80.7% of the subjects were infected after ending the zero-COVID-19 policy [16], all participants in that study were hospitalized patients, who might have relatively higher infection rates. In addition, we considered that children under 3 years age had the lowest chance to be infected with SARS-CoV-2 because almost all of the nursery schools were closed then and they were taken cared of at home. Thus, we calculated the sample size in this survey based an assumption that half (50%) of children had been infected with SARS-CoV-2. The participant size was calculated to be 384, with a confidence of 95% and a relative error of 10%. We enrolled 393 children who did not receive COVID-19 vaccination.

In addition, we included 114 children at the age of 3 − 6 years who had been vaccinated with at least two doses of inactivated COVID-19 vaccines (CoronaVac, Sinovac Life Sciences, or BBIBP-CorV, Sinopharm, both in Beijing, China) to serve as a comparison group. The COVID-19 vaccination history was collected from children parents or guardians when peripheral venous blood was taken. Additionally, the fact that all these 114 children attended kindergartens served as other evidence of COVID-19 vaccination, because children who did not receive COVID-19 vaccination were not permitted to attend kindergartens based on the rules of the zero-COVID-19 policy.

### Measurement of anti-SARS-CoV-2 IgG and IgM antibodies

Commercially available chemo-luminescence immunoassay kits (iFlash 3000 chemiluminescence immunoassay analyzer, Shenzhen YHLO Biotech, China) were used to measure IgG and IgM antibodies to SARS-CoV-2 respectively as previously described [19–21]. The kits contain purified recombinant nucleocapsid (N) and spike protein S1 subunit (S1) of SARS-CoV-2 synthesized in baculovirus. Therefore, the measured antibodies are directed against the N and S1 proteins of SARS-CoV-2 (anti-N/S1). Per the manufacturer’s instructions, a measurement equal to or higher than 10.0 arbitrary units (AU)/ml was considered positive, and that lower than 10.0 AU/mL was considered negative.

### Statistical analysis

Continuous data were presented as mean ± standard deviation or median (25th–75th percentile), and categorical data were presented as percentages. Characteristics of participants in different vaccination and sex groups were compared by unpaired Student’s t-test for age, by Mann-Whitney U-test for anti-N/S IgG titers, and by χ^2^ test for sex proportions. Seropositivity and Clopper-Pearson 95% confidence intervals (CI) were calculated, and compared in different groups by χ^2^ test. A two-sided P value of < 0.05 was considered as statistically significant. All statistical analyses were conducted using the SPSS 25.0 (version 25.0, SPSS, Chicago, IL, USA).

### Ethics considerations

This study was approved by the institutional review board (IRB) of Wujin Hospital of Changzhou city (No. 2023-SR-103). Written informed consent from was obtained from the parents/guardians of all children. The survey was carried out in accordance with the ethical standards of the 1964 Declaration of Helsinki and its later amendments.

## Results

Of the 393 unvaccinated children, 205 (52.2%) were boys and 188 (47.8%) were girls. Their mean age was 1.5 ± 0.6 years (median 1.2 years, 0.7–2.9), with 1.5 ± 0.6 in boys and 1.5 ± 0.6 in girls (t = 0.477, *P* = 0.634). Of these children, 369 (93.9%) had anti-N/S1 IgG ≥ 10 AU/ml, indicating anti-N/S1 IgG positive. The positive rate (93.7%, 192/205) of anti-N/S1 IgG in boys was comparable to that (94.1%, 177/188) in girls (χ^2^ = 0.041, *P* = 0.839) (Table 1). All 393 children with positive anti-N/S1 IgG had anti-N/S1 IgM < 10 AU/ml, indicating anti-N/S1 IgM negative, and none of 24 children with negative anti-N/S1 IgG was positive for anti-N/S1 IgM.

**Table 1:** Positive rate of anti-N/S1 IgG in children with different vaccination state

Vaccination state	No (%)*	Age, Years	Positive No	Positive rate, % (95% CI)
Unvaccinated	393	1.5 ± 0.6	369	93.9 (91.5–96.3)^†^
Boy	205 (52.2)	1.5 ± 0.6	192	93.7 (90.3–97.0)^§^
Girl	188 (47.8)	1.5 ± 0.6	177	94.1 (90.8–97.5)
Vaccinated^¶^	114	5.3 ± 0.9	112	98.2 (93.8–99.79)
Boy	55 (48.2)	5.3 ± 1.0	54	98.2 (90.3–99.95)^§^
Girl	59 (51.8)	5.4 ± 0.9	58	98.3 (90.9–99.96)

*The gender proportions between unvaccinated and vaccinated children had no statistically significant (χ^2^ = 0.543, *P* = 0.461)

^†^The difference in positive rate of anti-N/S1 IgG between unvaccinated and vaccinated children was not statistically significant (*χ*^*2*^ = 3.441, *P* = 0.064)

^§^The positive rate between boys and girls had no statistical difference (χ^2^ = 0.041, *P* = 0.839)

^¶^The children were injected with at least two doses of inactivated COVID-19 vaccines (CoronaVac, Sinovac Life Sciences, or BBIBP-CorV, Sinopharm, Beijing, China)

Of the 114 children who received COVID-19 vaccination, 55 (48.2%) were boys and 59 (51.8%) were girls. The gender proportions between the unvaccinated and vaccinated children had no statistical significance (χ^2^ = 0.543, *P* = 0.461). The mean age of vaccinated children was 5.3 ± 0.9 years (median 6.0 years, 3.4–6.7), with 5.3 ± 1.0 in boys and 5.4 ± 0.9 in girls (t = -0.564, *P* = 0.574). The positive rate (98.2%, 54/55) of anti-N/S1 IgG in boys was similar to that (98.3%, 58/59) in girls (χ^2^ = 0.001, *P* = 1.000) (Table 1). Of them, 112 (98.2%) were anti-N/S1 IgG positive, and none was anti-N/S1 IgM positive.

**Fig. 1:**
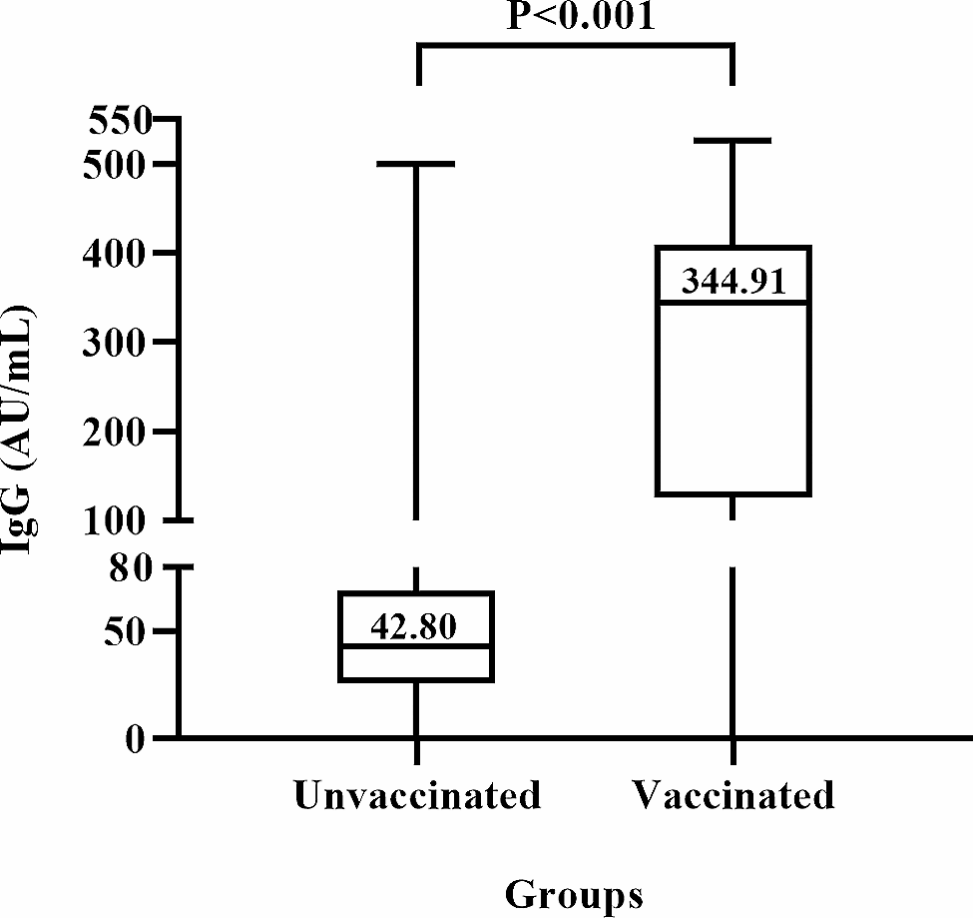
Comparison of titers of IgG antibody to nucleocapsid and subunit 1 of spike proteins of SARS-CoV-2 between vaccinated and unvaccinated children. Vaccinated children received at least two doses of inactivated COVID-19 vaccines (CoronaVac, Sinovac Life Sciences, or BBIBP-CorV, Sinopharm, Beijing, China)

**Fig. 2:**
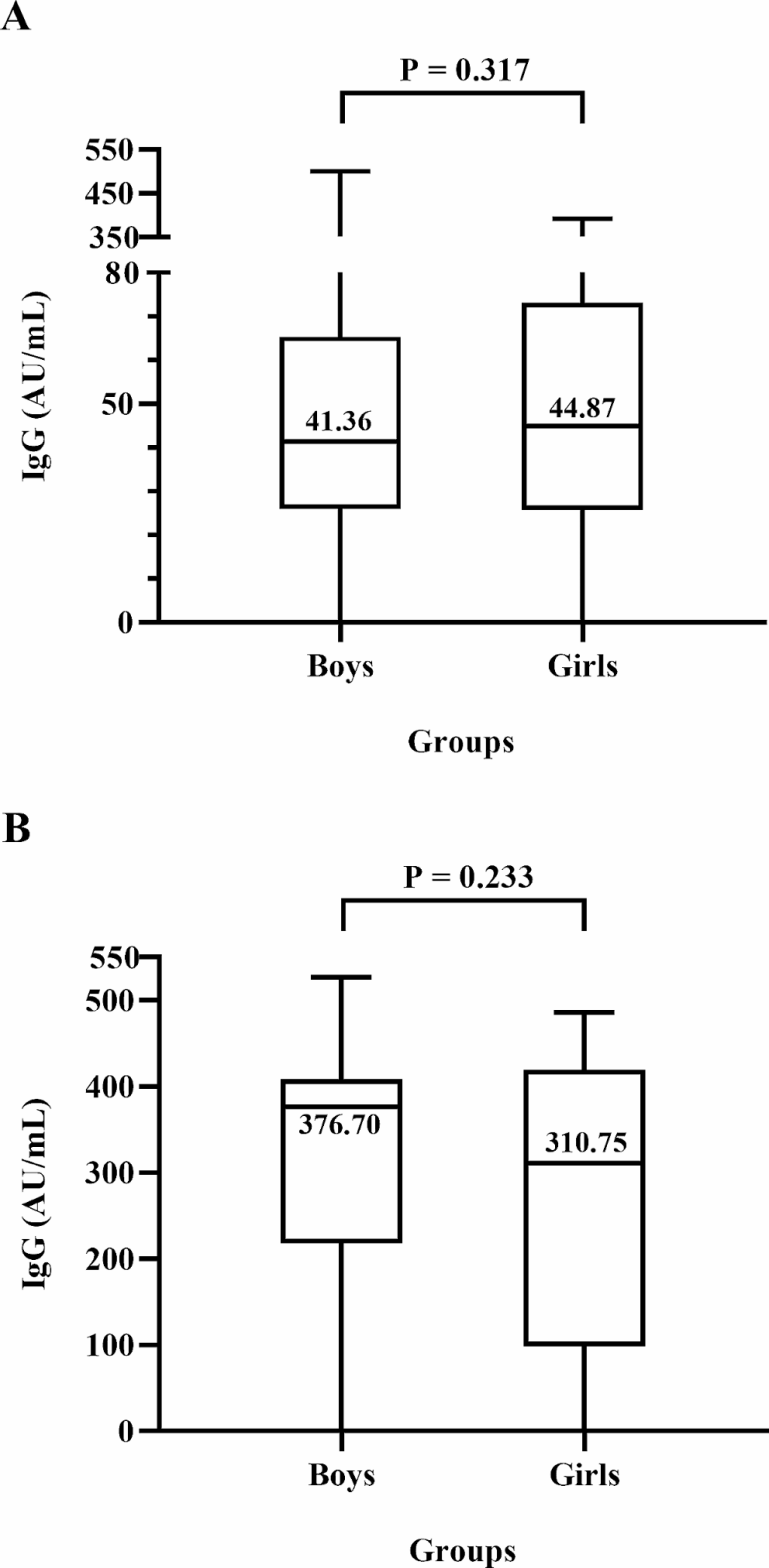
Comparison of titers of IgG antibody to nucleocapsid and subunit 1 of spike proteins of SARS-CoV-2 between boys and girls. (**A**) Unvaccinated children. (**B**) Vaccinated children

We further compared the median titers of anti-N/S1 IgG between the vaccinated and unvaccinated children (Fig. 1). The anti-N/S1 IgG titers among unvaccinated children were significantly lower than those in vaccinated children (42.80 vs. 344.90 AU/mL, Z = -10.509, *P* < 0.001). Additionally, Fig. 2 shows that the median titers between boys and girls had no statistically significant difference in either unvaccinated or vaccinated children (Z = -1.001, *P* = 0.317, and Z=-1.194, *P* = 0.233 respectively).

## Discussion

In this serosurvey, we found that as high as 93.9% (369/393) of the children who did not receive COVID-19 vaccination were positive for IgG antibodies against the N and S1 protein of SARS-CoV-2. Since these children were not immunized with COVID-19 vaccines and gastrointestinal adsorption of maternal anti-SARS-CoV-2 IgG into the blood circulation through breastfeeding is less likely [22], the anti-N/S1 IgG positivity confirmed the infection of SARS-CoV-2. As the children were enrolled from February 20 to April 10, 2023, and there were no cases of COVID-19 occurred among children in Changzhou city before December 7, 2022, the day of discontinuation of the zero-COVID-19 policy, the data in our study demonstrate that vast majority of the children were infected with SARS-CoV-2 shortly after discontinuation of the zero-COVID-19 policy.

In addition to the test of anti-N/S1 IgG in unvaccinated children, we also tested anti-N/S1 IgG in 114 children who were immunized with inactivated COVID-19 vaccines. The positive rate (98.2%) was somewhat higher than that (93.9%) in unvaccinated children (Table 1). Because these children were vaccinated with COVID-19 vaccines, the anti-N/S1 IgG in these children may be assumed to be elicited by the vaccination. However, these children had much higher (over 8 folds) anti-N/S1 IgG titers than unvaccinated children (Fig. 1). Duration of antibodies to SARS-CoV-2 after vaccination of inactivated COVID-19 vaccines is usually 3–5 months after the second vaccination course [23–26]. The COVID-19 vaccinations were completed in children during the autumn of 2021 (with two doses of vaccines) or during the spring of 2022 (with the booster dose). The interval between the last vaccination and blood collection was at least one year, as the children were included in the present study February 20 through April 10, 2023. Had they not been infected with SARS-CoV-2, they should have had undetectable or very low titers of anti-N/S1 IgG. Thus, the high titers of anti-N/S1 in the vaccinated children were likely caused by the brisk robust anamnestic antibody response, which may serve as the evidence of recent infection of SARS-CoV-2 in the majority, if not all, of these children.

It is interesting to note that none of the children with positive anti-N/S1 IgG antibody, including vaccinated and unvaccinated children, was positive for anti-N/S1 IgM antibody in the present study. Since the pandemic of COVID-19 occurred during the middle December 2022 to middle January 2023 and the children were enrolled between February 20 and April 10, 2023, the intervals between COVID-19 onset and blood collection were approximately from 5 to 15 weeks. Studies showed that IgM antibody directed to SARS-CoV-2 usually develops in the first week following the onset, peaks in the second and third week, and rapidly wanes from the fourth week [27–29], however, a considerable proportion of the patients at the early phase of COVID-19 outbreak in China during 2020 may still have detectable IgM antibodies to SARS-CoV-2 after 4 weeks [30], or even after recovery for several months and more than one year [31, 32]. Thus, the profile of decline of IgM antibody to SARS-CoV-2 observed in the present survey appeared to be different from that reported in the adult patients, which merits further study.

The high infection rate of SARS-CoV-2 in unvaccinated children observed in this study raises a question of whether these children who were not eligible for COVID-19 vaccination because of the young age should be vaccinated against SARS-CoV-2 when they are growing up for eligible for the vaccination. Clarification of this issue will be critical for making COVID-19 vaccination policy in the future. Another implication of the high SARS-CoV-2 infection rate in the children may help to estimate the overall incidence of SARS-CoV-2 infection occurred during the middle December 2022 to middle January 2023 in mainland China. Because of the unexpected overwhelming pandemics after ending the zero-COVID-19 policy, the overall incidence of SARS-CoV-2 infection was actually unknown. With a modeling transmission of SARS-CoV-2 Omicron based on the data of COVID-19 occurred in Shanghai, China, during the spring of 2022, it was estimated to have 112.2 million symptomatic cases (79.58 per 1,000 individuals) if the zero-COVID-19 policy had been discontinued [33]. However, based on the findings that over 90% children had the evidence of SARS-CoV-2 infection in the present survey, together with the results reported by others that 92.8% of children and 80.7% of individuals at all ages were infected [16, 34], we considered that at least over 80% of whole population in China experienced SARS-CoV-2 infection during the period of December 2022 to middle January 2023.

There two main limitations in this study. One is that the children were not randomly selected based on the children population. The other is that we did not collect the clinical data related to COVID-19 in the enrolled children. Thus, we are unable to define the proportions of symptomatic and asymptomatic COVID-19 cases and to compare the disease severity between the vaccinated and unvaccinated children. Clarification of this issue will be valuable to determine the COVID-19 vaccination policy in children.

In conclusion, this investigation demonstrates that over 90% of children were infected with SARS-CoV-2 after ending the zero-COVID-19 policy. Whether these children may be re-infected with SARS-CoV-2 in the future requires further study.

## Data Availability

The datasets used and/or analyzed during the current study are available from the corresponding authors on reasonable request.
